# Supplementary material for: Comparative analysis of the myoglobin gene in whales and humans reveals evolutionary changes in regulatory elements and expression levels
Source: PLoS One. 2023 Aug 29;18(8):e0284834. doi: 10.1371/journal.pone.0284834 (PMC10464968; doi:10.1371/journal.pone.0284834)

**S1 File. Supplement to Fig 1.**

**A** Flow chart of the experiments. Created under liscense from [BioRender.com](http://biorender.com/). See S9 Fig for photographs of differentiated C2C12 cells after 4 days.


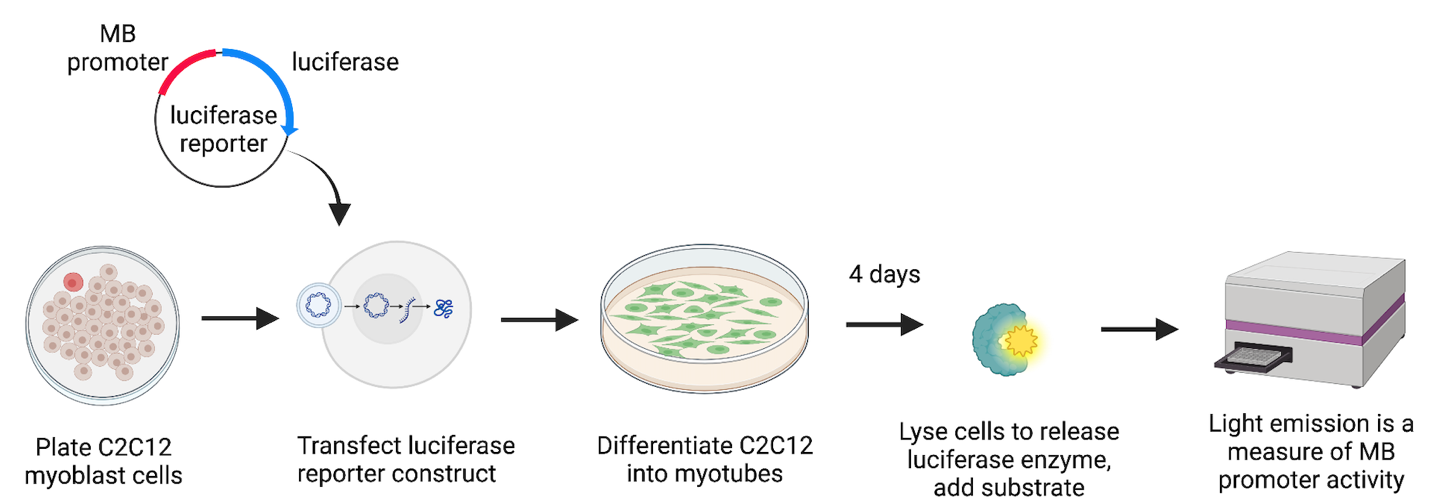


**B** Schematics of the internal deletions described in the text. To the right is the name of the deletion and in parentheses the nucleotides deleted in each case. Except for the two Ba410 derivatives, deletions are in the context of Ba710.


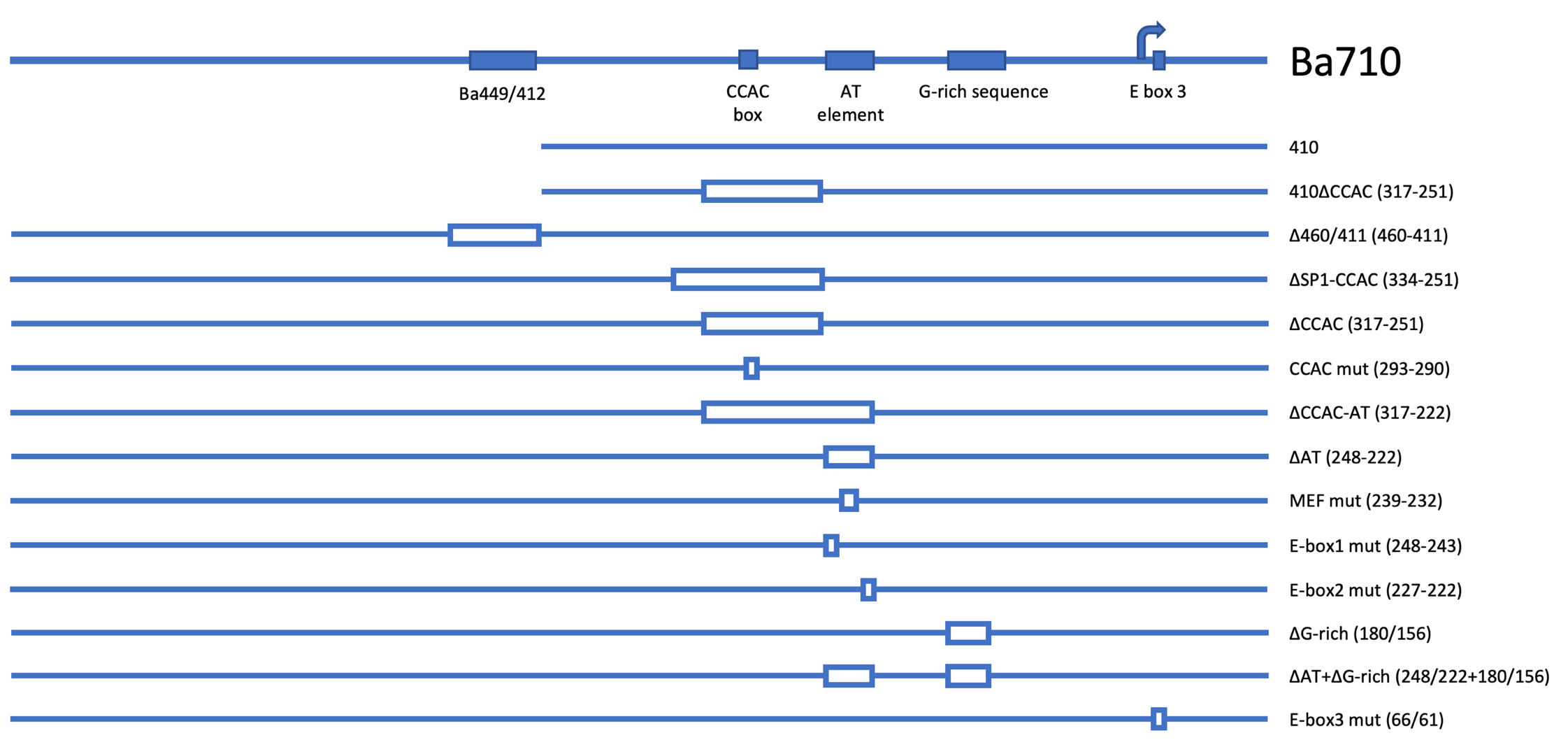

Supplement: S1 File — A Flow chart of the experiments. Created under license from BioRender.com. See S9 File for photographs of differentiated C2C12 cells after 4 days. B Schematics of the internal deletions described in the text. To the right is the name of the deletion and in parentheses the nucleotides deleted or otherwise affected in each case. Except for the two Ba410 derivatives, deletions are in the context of Ba710. (DOCX) [file pone.0284834.s001.docx]
