# Supplementary material for: Comparative analysis of the myoglobin gene in whales and humans reveals evolutionary changes in regulatory elements and expression levels
Source: PLoS One. 2023 Aug 29;18(8):e0284834. doi: 10.1371/journal.pone.0284834 (PMC10464968; doi:10.1371/journal.pone.0284834)
Supplement: S2 File — Data is expressed as F/R/Ba (see Materials and Methods). Four species not shown for simplicity in Table 1 or Fig 2 are included here: Balaenoptera musculus(Bm706), Megaptera novaeangliae (Mn704), Orca orcinus (Oo703), and Sus scrofa (Ss657). Data indicated by a superscript “x” is from clones inserted directly into the XhoI and NcoI sites of pGL4.10 without extraneous polylinker sequences. Data generated in C2C12 cells from the Wold lab are indicated by a superscript “w”; all other data was generated in C2C12 cells purchased directly from ATCC. The average of the seven cetacean species shown is 0.801; including the three Artiodactylan species yields an average of 0.782 and this number is used for Fig 2B (CetArt). “SEM” is standard error of the mean. (DOCX) [file pone.0284834.s002.docx]

**S2 File. Full data set for Table 1.**

Data is expressed as F/R/Ba (see Materials and Methods). Four species not shown for simplicity in Table 1 or Fig 2 are included here: *Balaenoptera musculus*(Bm706), *Megaptera novaeangliae* (Mn704), *Orca orcinus* (Oo703), and *Sus scrofa* (Ss657). Data indicated by a superscript “x” is from clones inserted directly into the XhoI and NcoI sites of pGL4.10 without extraneous polylinker sequences. Data generated in C2C12 cells from the Wold lab are indicated by a superscript “w”; all other data was generated in C2C12 cells purchased directly from ATCC. The average of the seven Cetacean species shown is 0.801; including the three Artiodactylan species yields an average of 0.782 and is used for Fig 2B (“CetArt”). “SEM” is standard error of the mean.

|  | GL4.10 (vector) | Ba710^x^ (minke) | Bm706^x^ (blue) | Mn704^x^ (humpback) | Er701^x^ (gray) | Oo703 (killer) | Dc706^x^ (dolphin) | Pp702 (porpoise) | Bt695 (cow) | Cc696^x^ (elk) | Ss657 (pig) | Ec675^x^ (horse) | Cf708 (dog) | Hs671^x^ (human) |
| --- | --- | --- | --- | --- | --- | --- | --- | --- | --- | --- | --- | --- | --- | --- |
|  | 0.015 | 1.018 | 0.703 ^w^ | 0.658^w^ | 0.716^w^ | 0.904 | 0.816 | 0.539 | 0.662 | 0.698 | 0.567 | 81.201 | 1.502 | 12.695 |
|  | 0.017 | 1.080 | 0.772 ^w^ | 0.756^w^ | 0.766^w^ | 0.793 | 1.068 | 0.532 | 1.032 | 0.723 | 0.637 | 80.837 | 1.353 | 11.254 |
|  | 0.022 | 0.971 | 0.845 ^w^ | 0.666^w^ | 0.847^w^ |  | 0.810 | 0.782 | 0.734 | 0.658 | 0.671 | 65.782 | 2.051 | 10.605 |
|  | 0.065 | 1.050 | 0.860 ^w^ | 0.693^w^ | 0.848^w^ |  | 0.743 | 0.916 | 0.981 | 0.813 | 0.581 | 68.971 | 1.443 | 11.488 |
|  | 0.017 | 1.069 |  | 0.545 |  |  |  | 0.425^x^ | 0.983 | 0.702 |  |  |  | 17.700 |
|  |  |  |  |  |  |  |  | 0.476^x^ |  |  |  |  |  |  |
| n | 5 | 5 | 4 | 5 | 4 | 2 | 4 | 6 | 5 | 5 | 4 | 4 | 4 | 5 |
| mean | 0.027 | 1.037 | 0.795 | 0.664 | 0.794 | 0.848 | 0.859 | 0.612 | 0.878 | 0.719 | 0.614 | 74.198 | 1.587 | 12.748 |
| SEM | 0.010 | 0.020 | 0.036 | 0.034 | 0.032 | 0.056 | 0.071 | 0.079 | 0.075 | 0.026 | 0.024 | 3.992 | 0.158 | 1.283 |
