# Supplementary material for: Comparative analysis of the myoglobin gene in whales and humans reveals evolutionary changes in regulatory elements and expression levels
Source: PLoS One. 2023 Aug 29;18(8):e0284834. doi: 10.1371/journal.pone.0284834 (PMC10464968; doi:10.1371/journal.pone.0284834)
Supplement: S3 File — A Alignment of the AT element (E-box1—Mef-2—E-box2): Hash marks indicate identity with the human (Hs) sequence. Most cetaceans (Mn, Er, Bm, Dc, Oo) and the artiodactylans (Bt, Cc) are identical to Ba through the AT element; Pp has a variant E-box1 (CACATG instead of CACCTG). B Alignment of E-box3 region: The Odontoceti, Oo (Orca orcinus), Dc (Delphinus capensis), Tt (Tursiops truncatus), and Pp (Phocoena phocoena) vary from the CAGCTG present in the other species presented. The bottlenose dolphin, T. truncatus, is included because no published sequence is available for the common dolphin, D. capensis. Mm: Mus musculus. The variant sequence in the Odontoceti is not predicted by rVISTA to bind any of the transcription factors shown in Fig 3C. C Full data set, average of duplicate wells, normalized as F/R/Ba. Equal variances confirmed, based on homogeneity of variances test (indicated here and below with an asterisk on the construct name). Analysis of variance (ANOVA) confirms a statistical difference between the samples (F(5,24) = 24.955, p <0.001). ANOVA was followed by the post-hoc Tukey HSD test [35]. D Tukey test data for Fig 3B. (DOCX) [file pone.0284834.s003.docx]

**S3 File. Supporting information for Fig 3.**

**A** Alignment of the AT element (E-box1--Mef-2--E-box2): Hash marks indicate identity with the human (Hs) sequence. Most Cetaceans (Mn, Er, Bm, Dc, Oo) and the Artiodactylans (Bt, Cc) are identical to Ba through the AT element; Pp has a variant E-box1 (CACATG instead of CACCTG).

Ba CCCACAATGGCACCTGCCTCAAAATAGCTTC-CATGTGAGGGCCAGAG

||||||||||||||||| ||||||||||| ||||||||||| ||||

Pp cccccaatggcacatgcctcaaaatagcttc-catgtgagggctagag

|| ||||||||| |||| ||||||||||| ||||||||||||||||

Bt CCCACAATGGCACCTGCCTCAAAATAGCTTC-CATGTGAGGGCTAGAG

||||||||||||||||| ||||||||||| ||||||||||||||||

Ec CCCCCAATGACACCTGCCCCAAAATAGCTCC-CATGTGAGGGCTAGAG

|| ||||| ||||||||| ||||||||| | ||||||||||||||||

Cf CCCACAATGGCACCTGCCCCAAAATAGCTTC-CATGTGAGAGCTACAG

|||||||||||||||||| ||||||||||| |||||||| |||| ||

Hs GCCACAATGGCACCTGCCCTAAAATAGCTTCCCATGTGAGGGCTAGAG

**E-box1 Mef-2 E-box2**

**B** Alignment of E-box3 region: The Odontoceti, Oo (*Orca orcinus*), Dc (*Delphinus capensis*), Tt (*Tursiops truncatus*), and Pp (*Phocoena phocoena*) vary from the CAGCTG present in the other species presented. The bottlenose dolphin, *T. truncatus*, is included because no published sequence is available for the common dolphin, *D. capensis*. Mm: *Mus musculus*.

The variant sequence in the Odontoceti is not predicted by rVISTA to bind any of the transcription factors shown in Fig 3C.

Ba CCTCAAACCC**CAGCTG**TCGGAGCCAG

Bm CCTCAAACCC**CAGCTG**TCGGAGCCAG

Er CCTCAAACCC**CAGCTG**TCGGAGCCAG

Mn CCTCAAACCC**CAGCTG**TCGGAGCCAG

Oo CCTCAGACCCCAGCGGTCGGAGCCAG

Dc CCTCAGACCCCAGCGGTCGGAGCCAG

Tt cctcagaccccagcggtcggagccag

Pp cctcaaaccccagcggtcggagccag

Ec CCTCAAACCC**CAGCTG**TCAGGGCCAG

Cf TCTCAAACCC**CAGCTG**TCAAAGCCAG

Hs CCTCAAACCC**CAGCTG**TTGGGGCCAG

Mm CATTCAGCAC**CAGCTG**CCACTCCCCA

**C** Full data set, average of duplicate wells, normalized as F/R/Ba. Equal variances confirmed, based on homogeneity of variances test (indicated here and below with an asterisk on the construct name). Analysis of variance (ANOVA) confirms a statistical difference between the samples (*F*(5,24) = 24.955, *p* <0.001). ANOVA was followed by the post-hoc Tukey HSD test [35].

|  | ATswap* | MEFmut | ΔAT* | Ebox1mut | Ebox2mut* | Ebox3mut* |
| --- | --- | --- | --- | --- | --- | --- |
|  | 0.995 | 0.816 | 0.639 | 0.929 | 0.854 | 0.619 |
|  | 0.899 | 0.831 | 0.622 | 0.740 | 0.851 | 0.638 |
|  | 1.048 | 0.967 | 0.754 | 0.950 | 0.928 | 0.563 |
|  | 1.111 | 0.972 | 0.732 | 0.927 | 0.903 | 0.499 |
|  | 0.980 | 0.839 | 0.637 | 0.946 | 1.031 | 0.541 |
| n | 5 | 5 | 5 | 5 | 5 | 5 |
| mean | 1.007 | 0.885 | 0.677 | 0.899 | 0.913 | 0.572 |
| SEM | 0.035 | 0.035 | 0.028 | 0.040 | 0.033 | 0.026 |

**D** Tukey test data for Fig 3B

| Tukey's multiple comparisons test | Mean Diff. | 95.00% CI of diff. | Below threshold? | Summary | Adjusted P Value |
| --- | --- | --- | --- | --- | --- |
| **ATswap vs. MEFmut** | -0.1219 | -0.2661 to 0.02237 | No | ns | 0.133 |
| **ATswap vs. ΔAT** | -0.3299 | -0.4741 to -0.1856 | Yes | **** | <0.0001 |
| **ATswap vs. Ebox1mut** | 0.1082 | -0.03601 to 0.2525 | No | ns | 0.225 |
| **ATswap vs. Ebox2mut** | 0.09337 | -0.05086 to 0.2376 | No | ns | 0.370 |
| **ATswap vs. Ebox3mut** | 0.4347 | 0.2905 to 0.5789 | Yes | **** | <0.0001 |
| MEFmut vs. ∆AT | 0.2080 | 0.06379 to 0.3522 | Yes | ** | 0.0020 |
| MEFmut vs. Ebox1mut | -0.01363 | -0.1579 to 0.1306 | No | ns | 0.9997 |
| MEFmut vs. Ebox2mut | -0.02849 | -0.1727 to 0.1157 | No | ns | 0.9892 |
