# Supplementary material for: Comparative analysis of the myoglobin gene in whales and humans reveals evolutionary changes in regulatory elements and expression levels
Source: PLoS One. 2023 Aug 29;18(8):e0284834. doi: 10.1371/journal.pone.0284834 (PMC10464968; doi:10.1371/journal.pone.0284834)
Supplement: S4 File — A Multiple species alignment of the CCAC-box region: The 10 nt conserved core of the CCAC-box [15] encompasses Hs289/280 and Ba297/288. Hash marks indicate identity with the human (Hs) sequence. Sp1 binding (bold type) to the Ba sequence is robustly predicted by rVISTA, LASAGNA, and MATCH, and to the Hs sequence by rVISTA, MATCH and JASPAR; Sp1 binding is also predicted by rVISTA for Pp, Ss, and Cf, but not for Bt or Ec. Other transcription factors that are predicted by rVISTA (but not necessarily conserved) and are expressed in muscle are shown: AP2 (asterisks), MAZR (colon), MYOD (wavy underline), PPARalpha (double dashed underline), and USF (underline). B F Full data set, average of duplicate wells, normalized as F/R/Ba. Equal variances confirmed, based on homogeneity of variances test. ANOVA confirms a statistical difference between the samples (F(7,35) = 99.449, p <0.001). ANOVA was followed by the post-hoc Tukey HSD test. C Tukey test for Fig 4B and accompanying text. D Full data set for Ba410 and Ba410ΔCCAC, average of duplicate wells, normalized as F/R/Ba. Variances between these two samples are not equal. (DOCX) [file pone.0284834.s004.docx]

**S4 File. Supporting information for Fig 4.**

**A** Multiple species alignment of the CCAC-box region: The 10 nt conserved core of the CCAC-box [15] encompasses Hs289/280 and Ba297/288. Hash marks indicate identity with the human (Hs) sequence. Sp1 binding (bold type) to the Ba sequence is robustly predicted by rVISTA, LASAGNA, and MATCH, and to the Hs sequence by rVISTA, MATCH and JASPAR; Sp1 binding is also predicted by rVISTA for Pp, Ss, and Cf, but not for Bt or Ec. Other transcription factors that are predicted by rVISTA (but not necessarily conserved) and are expressed in muscle are shown: AP2 (asterisks), MAZR (colon), MYOD (wavy underline), PPARalpha (double dashed underline), and USF (underline).

297* *288

Ba TCCACACACACA**G-CTCCT-CCCCACCCCC**CCAACCTGACGCCTGAGTT

===================

||||||| |||| || |||||||||| || ||||||| |

Pp TCCACAC**ACACAG-CCCCTCCCCCCCC**----AAACCTGAGGCCTGAGTT

::: :::::::::::::

=========== ========

||||||| |||| || |||| || || |||||||| |

Bt CGCACACACAACCCCACCACCACCACACCC-------GTGACCTGAGTT

====== ======

||||| || |||| | |||| ||| ||| |||||| |

Ss TCCACCCACACA---A--**A--CCCACCCCC**-------GTGGCCTGAGTT

= ========= =========

******* ******

||||| | |||| | | ||||||||| |||||||||| |

Ec TCCACACACACA---------GCCACCCCCC-----TGCGGCGTCCTAC

:: :::::::::: :

********** ******

||||||| |||| |||||||| || ||| |

Cf GCCACATACACA---AACAC**CCCCACGCCCC-----TG**TGGCCTGAGTT

========= ==========

||||| |||| | || |||||| ||| ||||||||||| |

Hs TCCACACGCACA---AC**CA-CCCCACCCCC------T**GTGGCCTGAGCTE=∆CCAC-AT,

::: ::::::::::

========= ==========

289* *280

**B** Full data set, average of duplicate wells, normalized as F/R/Ba. Equal variances confirmed, based on homogeneity of variances test. ANOVA confirms a statistical difference between the samples (*F*(7,35) = 99.449, *p* <0.001). ANOVA was followed by the post-hoc Tukey HSD test.

|  | CCACmut* | ΔCCAC* | ΔSP1-CCAC* | ΔCCAC-AT* | CCACswap* | CCAC+ATswap* |
| --- | --- | --- | --- | --- | --- | --- |
|  | 0.841 | 1.056 | 0.980 | 0.553 | 1.112 | 1.821 |
|  | 0.942 | 1.063 | 0.832 | 0.608 | 1.285 | 1.742 |
|  | 0.807 | 1.189 | 0.890 | 0.663 | 1.096 | 1.665 |
|  | 0.850 | 1.136 | 0.870 | 0.566 | 1.203 | 1.646 |
|  | 0.811 | 1.056 | 1.004 | 0.569 | 1.237 | 1.669 |
|  |  | 1.033 |  |  |  |  |
|  |  | 1.218 |  |  |  |  |
|  |  | 1.017 |  |  |  |  |
| n | 5 | 8 | 5 | 5 | 5 | 5 |
| mean | 0.850 | 1.096 | 0.915 | 0.592 | 1.187 | 1.708 |
| SEM | 0.024 | 0.027 | 0.033 | 0.020 | 0.036 | 0.033 |

**C** Tukey tests for Fig 4B and accompanying text.

| Tukey's multiple comparisons test | Mean Diff. | 95.00% CI of diff. | Below threshold? | Summary | Adjusted P Value |
| --- | --- | --- | --- | --- | --- |
| **Ba ΔSP1-CCAC vs. Ba CCACmut** | -0.06522 | -0.1988 to 0.06837 | No | ns | 0.670 |
| **Ba ΔSP1-CCAC vs. Ba ΔCCAC** | 0.1807 | 0.06031 to 0.3012 | Yes | ** | 0.001 |
| **Ba ΔSP1-CCAC vs. Ba ΔCCAC-AT** | 0.3234 | 0.1898 to 0.4570 | Yes | **** | <0.0001 |
| **Ba ΔSP1-CCAC vs. CCAC swap** | -0.2715 | -0.4051 to -0.1379 | Yes | **** | <0.0001 |
| **Ba ΔSP1-CCAC vs. CCAC+AT swap** | -0.7931 | -0.9267 to -0.6595 | Yes | **** | <0.0001 |
| **CCAC swap vs. CCAC+AT swap** | -0.5217 | -0.6553 to -0.3881 | Yes | **** | <0.0001 |

Tukey test comparing Ba ∆AT, Ba ∆CCAC, and Ba ∆CCAC-AT. See S3C File for the ∆AT data used.

| Tukey's multiple comparisons test | Mean Diff. | 95.00% CI of diff. | Below threshold? | Summary | Adjusted P Value |
| --- | --- | --- | --- | --- | --- |
| **Ba ΔAT vs. Ba ΔCCAC** | -0.4191 | -0.5148 to -0.3235 | Yes | **** | <0.0001 |
| **Ba ΔAT vs. Ba ΔCCAC-AT** | 0.08498 | -0.02112 to 0.1911 | No | ns | 0.1277 |
| **Ba ΔCCAC vs. Ba ΔCCAC-AT** | 0.5041 | 0.4085 to 0.5997 | Yes | **** | <0.0001 |

**D** Full data set for Ba410 and Ba410ΔCCAC, average of duplicate wells, normalized as F/R/Ba. Variances between these two samples are not equal.

|  | 410* | 410ΔCCAC* |
| --- | --- | --- |
|  | 0.751 | 0.647 |
|  | 0.781 | 0.707 |
|  | 0.770 | 0.958 |
|  | 0.652 | 0.854 |
|  | 0.735 | 0.954 |
|  | 0.756 |  |
| n | 6 | 5 |
| mean | 0.741 | 0.824 |
| SEM | 0.009 | 0.064 |
