# Supplementary material for: Comparative analysis of the myoglobin gene in whales and humans reveals evolutionary changes in regulatory elements and expression levels
Source: PLoS One. 2023 Aug 29;18(8):e0284834. doi: 10.1371/journal.pone.0284834 (PMC10464968; doi:10.1371/journal.pone.0284834)
Supplement: S5 File — A Full data set, average of duplicate wells, normalized as F/R/Ba. Equal variances confirmed, based on homogeneity of variances test. ANOVA confirms a statistical difference between the samples (F(3,15) = 16.109, p <0.001). ANOVA was followed by the post-hoc Tukey HSD test. The data in heavy boxes is derived from four transfected plates, allowing direct comparison of the activities of Hs671, Hs ΔAT, Hs ΔCCAC, and Hs ΔG-rich; note that although the data for Hs ΔG-rich is not statistically different from that for Hs671, in each case the Hs ΔG-rich samples are lower than the Hs671 values. The same Hs671 data shown here was included in the data set in Table 1. B Tukey test for Fig 5. (DOCX) [file pone.0284834.s005.docx]

**S5 File. Supporting information for Fig 5.**

**A** Full data set, average of duplicate wells, normalized as F/R/Ba. Equal variances confirmed, based on homogeneity of variances test. ANOVA confirms a statistical difference between the samples (*F*(3,15) = 16.109, *p* <0.001). ANOVA was followed by the post-hoc Tukey HSD test. The data in heavy boxes is derived from four transfected plates, allowing direct comparison of the activities of Hs671, HsΔAT, HsΔCCAC, and HsΔG-rich; note that although the data for HsΔG-rich is not statistically different from that for Hs671, in each case the HsΔG-rich samples are lower than the Hs671 values.

The same Hs671 data shown here was included in the data set in Table 1.

| plate | Hs671* | HsΔAT* | HsΔCCAC* | HsΔG-rich* |
| --- | --- | --- | --- | --- |
| 1 | 17.700 | 4.432 | 11.928 | 12.910 |
| 2 | 11.488 | 3.094 | 7.615 | 9.532 |
| 3 | 10.605 | 2.233 | 7.399 | 8.631 |
| 4 | 11.254 | 2.785 | 6.175 | 10.014 |
|  | 12.695 |  | 8.038 | 8.759 |
| n | 5 | 4 | 5 | 5 |
| mean | 12.748 | 3.136 | 8.231 | 9.969 |
| SEM | 1.283 | 0.467 | 0.975 | 0.778 |

**B** Tukey test for Fig 5.

| Tukey's multiple comparisons test | Mean Diff. | 95.00% CI of diff. | Below threshold? | Summary | Adjusted P Value |
| --- | --- | --- | --- | --- | --- |
| **Hs671 vs. HsΔAT** | 9.61 | 5.54 to 13.7 | Yes | **** | <0.0001 |
| **Hs671 vs. HsΔCCAC** | 4.52 | 0.675 to 8.36 | Yes | * | 0.019 |
| **Hs671 vs. HsΔG-rich** | 2.78 | -1.06 to 6.62 | No | ns | 0.203 |
