# Supplementary material for: Comparative analysis of the myoglobin gene in whales and humans reveals evolutionary changes in regulatory elements and expression levels
Source: PLoS One. 2023 Aug 29;18(8):e0284834. doi: 10.1371/journal.pone.0284834 (PMC10464968; doi:10.1371/journal.pone.0284834)
Supplement: S6 File — A Full data set, average of duplicate wells, normalized as F/R/Ba. Equal variances confirmed, based on homogeneity of variances test. ANOVA confirms a statistical difference between the samples (F(3,15) = 151.582, p <0.001). ANOVA was followed by the post-hoc Tukey HSD test. B Full data set, average of duplicate wells, normalized as F/R/Ba. The data in heavy boxes is derived from three transfected plates, allowing direct comparison of the activities of Ba710, Ba925, and Ba3kb-925. Equal variances confirmed, based on homogeneity of variances test. Analysis by ANOVA fails to find a statistical difference between the samples (F(3,15) = 1.152, p = 0.360). ANOVA was followed by the post-hoc Tukey HSD test. C Tukey test for Fig 6C. D Left: Dot plot alignment (PipMaker [98]) of 1000 nt of mouse (Mus musculus, Mm) MB sequence 5’ of the ATG (Y-axis) against 1000 nt of human MB sequence 5 of the ATG (X-axis). The diagonal dashed line plots similarities between the two sequences using default settings. The 5’ end of the similarities occurs at nts Hs474 and Mm422. The HindIII site at Hs444 is shown for reference. Right: Dot plot alignment of 1000 nt of Ba MB sequence 5’ of the ATG (Y-axis) against 1000 nt of Hs MB sequence 5’ of the ATG (X-axis) shown for comparison. E Multiple species alignment of conserved sequences at Ba460/411. Vertical hash marks indicate identity with the Hs sequence. The HindIII site (AAGCTT) is bold for reference. Transcription factor sites conserved (rVISTA) with humans and expressed in muscle are indicated: ATF3 (underlined), MEIS1-TGIF (double underline), CP2 (asterisk), NFE2L1/TCF11 (dashed line), TTF1 (dotted). (DOCX) [file pone.0284834.s006.docx]

**S6 File. Supporting information for Fig 6.**

**A** Full data set, average of duplicate wells, normalized as F/R/Ba. Equal variances confirmed, based on homogeneity of variances test. ANOVA confirms a statistical difference between the samples (*F*(3,15) = 151.582, *p* <0.001). ANOVA was followed by the post-hoc Tukey HSD test.

|  | ΔGAGA* | ΔG-rich* | ΔAT+G-rich* | BaΔ460/411* |
| --- | --- | --- | --- | --- |
|  | 0.943 | 0.450 | 0.302 | 0.841 |
|  | 1.069 | 0.391 | 0.320 | 0.695 |
|  | 1.101 | 0.486 | 0.265 | 0.726 |
|  | 1.062 | 0.346 | 0.331 | 0.713 |
|  |  | 0.413 | 0.303 | 0.848 |
| n | 4 | 5 | 5 | 5 |
| mean | 1.044 | 0.417 | 0.304 | 0.764 |
| SEM | 0.035 | 0.024 | 0.011 | 0.033 |

**B** Full data set, average of duplicate wells, normalized as F/R/Ba. The data in heavy boxes is derived from three transfected plates, allowing direct comparison of the activities of Ba710, Ba925, and Ba3kb-925. Equal variances confirmed, based on homogeneity of variances test. Analysis by ANOVA fails to find a statistical difference between the samples (*F*(3,15) = 1.152, *p* =0.360). ANOVA was followed by the post-hoc Tukey HSD test.

| plate | 511 | 710x* | Ba925* | Ba3kb-925* |
| --- | --- | --- | --- | --- |
|  | 1.274 | 1.018 | 0.900 |  |
|  | 0.942 | 1.080 | 1.270 |  |
| 1 | 1.036 | 0.971 | 1.211 | 1.078 |
| 2 | 1.040 | 1.050 | 1.133 | 1.274 |
| 3 | 1.067 | 1.069 | 1.098 | 1.130 |
|  | 0.978 |  |  |  |
| n | 6 | 5 | 5 | 3 |
| mean | 1.056 | 1.037 | 1.122 | 1.161 |
| SEM | 0.047 | 0.020 | 0.063 | 0.058 |

**C** Tukey test for Fig 6C.

| Tukey's multiple comparisons test | Mean Diff. | 95.00% CI of diff. | Below threshold? | Summary | Adjusted P Value |
| --- | --- | --- | --- | --- | --- |
| **Ba710 vs. ΔG-rich** | -0.6203 | -0.7796 to -0.4611 | Yes | **** | <0.0001 |
| **Ba710 vs. ΔAT+ΔG-rich** | -0.7332 | -0.8924 to -0.5739 | Yes | **** | <0.0001 |
| **Ba710 vs. Δ460/411** | -0.2730 | -0.4322 to -0.1138 | Yes | *** | <0.001 |
| **Ba710 vs. Ba925** | -0.08471 | -0.2439 to 0.07451 | No | ns | 0.572 |
| **Ba710 vs. Ba3kb-925** | -0.1234 | -0.3072 to 0.06049 | No | ns | 0.328 |

**D** Left: Dot plot alignment (PipMaker [98]) of 1000 nt of mouse (*Mus musculus*, Mm) MB sequence 5’ of the ATG (Y-axis) against 1000 nt of human MB sequence 5 of the ATG (X-axis). The diagonal dashed line plots similarities between the two sequences using default settings. The 5’ end of the similarities occurs at nts Hs474 and Mm422. The HindIII site at Hs444 is shown for reference.

Right: Dot plot alignment of 1000 nt of Ba MB sequence 5’ of the ATG (Y-axis) against 1000 nt of Hs MB sequence 5’ of the ATG (X-axis) shown for comparison.


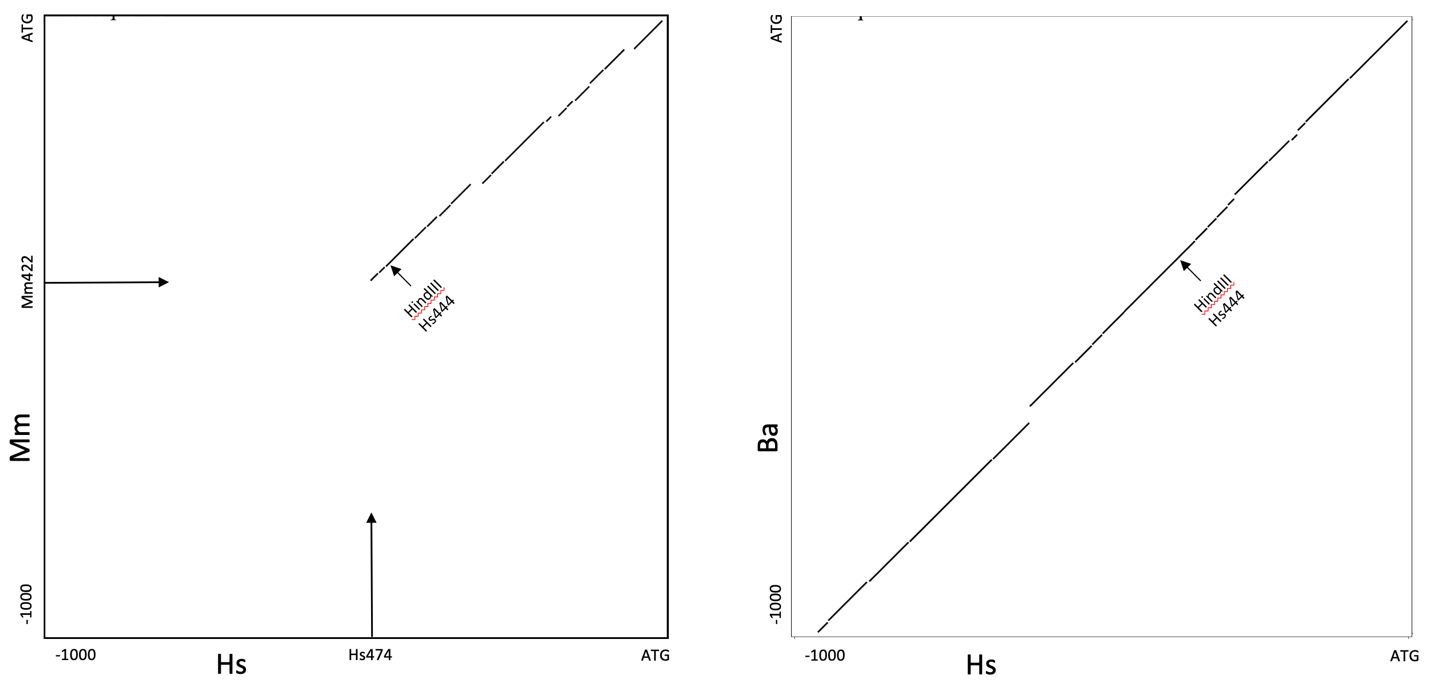


**E** Multiple species alignment of conserved sequences at Ba460/411. Vertical hash marks indicate identity with the Hs sequence. The HindIII site (AAGCTT) is bold for reference.

Transcription factor sites conserved (rVISTA) with humans and expressed in muscle are indicated: ATF3 (underlined), MEIS1-TGIF (double underline), CP2 (asterisk), NFE2L1/TCF11 (dashed line), TTF1 (dotted).

*Ba460 Ba411*

Ba CCCTGG-CTAATAAACATGACAGGTCCTCTTGGGATGGCTGACAGCAGGG

***********

|| | || | ||||||||||||||||||||||| ||||||| |||||| 84%

Pp CCCTGG-CTAATAAACATGACAGGTCCTCTTGGGATGGCTGACAGCAGGG

***********

|| | || | ||||||||||||||||||||||| ||||||| |||||| 84%

Bt CCCTGGGCTTAGAGATATGACAGGTCCTCTTGGGGTGGCTGACAGCAGGG

|| | |||||| | |||||||||||||||||| ||||||| |||||| 82%

Ss TCCAGG-CTTAGAAACATGACAGGTCCTCTTGAGAAGGATGACAGCAGGG

||| | ||||||||||||||||||||||||| || || |||| |||||| 86%

Ec -CC**AAG-CTT**GGGAACATGACAATTCCTCTGGGGGTGGCTGACAGCAGGG

||||| ||| | ||||||||| |||||| ||| ||||||| |||||| 80%

Cf CCTGAG-CTTGAAAACATGACAGCTCCTCCTGGGATGGCTGACAACGGGG

-------------

| || ||| ||||||||||| ||||| ||||| ||||||| | ||| 76%

Hs GCC**AAG-CTT**AGAAACATGACAGGTCCTCTTGGGAGGGCTGACCGCAGGG

------------- ***********

||||| ||||||||||||||| |||| |||| || ||| | || 72%

Mm CCC**AAG-CTT**AGAAACATGACAAGTCCCTGTGGGGCAGCAGACAGGGGGA
