# Supplementary material for: Comparative analysis of the myoglobin gene in whales and humans reveals evolutionary changes in regulatory elements and expression levels
Source: PLoS One. 2023 Aug 29;18(8):e0284834. doi: 10.1371/journal.pone.0284834 (PMC10464968; doi:10.1371/journal.pone.0284834)
Supplement: S7 File — (DOCX) [file pone.0284834.s007.docx]

**S7 File. Sources for transcription factors’ expression in muscle.**

URLs from: <https://www.genecards.org> [34]and [www.proteinatlas.org](http://www.proteinatlas.org) [96] are indicated.

AP2: <https://www.genecards.org/cgi-bin/carddisp.pl?gene=TFAP2A&keywords=AP2#expression>,

AP4: <https://www.genecards.org/cgi-bin/carddisp.pl?gene=TFAP4&keywords=AP4>, <https://www.proteinatlas.org/ENSG00000090447-TFAP4/tissue>

ATF3: <https://www.genecards.org/cgi-bin/carddisp.pl?gene=ATF3&keywords=ATF3>, <https://www.proteinatlas.org/ENSG00000162772-ATF3/tissue>

BHLHE40/DEC1: <https://www.genecards.org/cgi-bin/carddisp.pl?gene=BHLHE40>, <https://www.proteinatlas.org/ENSG00000134107-BHLHE40/tissue>30406

CP2: <https://www.genecards.org/cgi-bin/carddisp.pl?gene=TFCP2&keywords=CP2>, <https://www.proteinatlas.org/ENSG00000135457-TFCP2/tissue>.

LMO2: <https://www.genecards.org/cgi-bin/carddisp.pl?gene=LMO2>, <https://www.proteinatlas.org/ENSG00000135363-LMO2/tissue>

MEF2A: <https://www.genecards.org/cgi-bin/carddisp.pl?gene=MEF2A&keywords=MEF2A>, <https://www.proteinatlas.org/ENSG00000068305-MEF2A/tissue>

MEIS1: <https://www.genecards.org/cgi-bin/carddisp.pl?gene=MEIS1&keywords=MEIS1>, <https://www.proteinatlas.org/ENSG00000143995-MEIS1/tissue> .

MITF: <https://www.genecards.org/cgi-bin/carddisp.pl?gene=MITF&keywords=MITF>, <https://www.proteinatlas.org/ENSG00000187098-MITF/tissue>

MYF6: <https://www.genecards.org/cgi-bin/carddisp.pl?gene=MYF6&keywords=MYF6#expression>, <https://www.proteinatlas.org/ENSG00000111046-MYF6/tissue>

MYOD: <https://www.genecards.org/cgi-bin/carddisp.pl?gene=MYOD1&keywords=MyoD>, <https://www.proteinatlas.org/ENSG00000129152-MYOD1/tissue>

MYOG (myogenin): <https://www.genecards.org/cgi-bin/carddisp.pl?gene=MYOG&keywords=myogenin>, <https://www.proteinatlas.org/ENSG00000122180-MYOG/tissue>

NF1A: <https://www.genecards.org/cgi-bin/carddisp.pl?gene=NFIA&keywords=NF1A>, <https://www.proteinatlas.org/ENSG00000162599-NFIA/tissue>

NFE2L1/TCF11: <https://www.genecards.org/cgi-bin/carddisp.pl?gene=NFE2L1&keywords=TCF11> , <https://www.proteinatlas.org/ENSG00000082641-NFE2L1/tissue>

PATZ1/MAZR: <https://www.genecards.org/cgi-bin/carddisp.pl?gene=PATZ1>, <https://www.proteinatlas.org/ENSG00000100105-PATZ1/tissue>

PPARalpha: <https://www.genecards.org/cgi-bin/carddisp.pl?gene=PPARA&keywords=PPAR,alpha>, <https://www.proteinatlas.org/ENSG00000186951-PPARA/tissue>,

TCF3/E2A (E12, E47): <https://www.genecards.org/cgi-bin/carddisp.pl?gene=TCF3&keywords=E12,E2A>, <https://www.proteinatlas.org/ENSG00000071564-TCF3/tissue>

TCF12/HEB: <https://www.genecards.org/cgi-bin/carddisp.pl?gene=TCF12&keywords=HEB>, <https://www.proteinatlas.org/ENSG00000140262-TCF12/tissue>

TFE3: <https://www.genecards.org/cgi-bin/carddisp.pl?gene=TFE3&keywords=TFE>, https://www.proteinatlas.org/ENSG00000068323-TFE3/tissue

TGIF1: <https://www.genecards.org/cgi-bin/carddisp.pl?gene=TGIF1&keywords=TGIF1>, <https://www.proteinatlas.org/ENSG00000177426-TGIF1/tissue>.

UBP1/LBP1: <https://www.genecards.org/cgi-bin/carddisp.pl?gene=UBP1>, <https://www.proteinatlas.org/ENSG00000153560-UBP1/tissue>

USF: <https://www.genecards.org/cgi-bin/carddisp.pl?gene=USF1>, <https://www.proteinatlas.org/ENSG00000158773-USF1/tissue>

ZIC1: <https://www.genecards.org/cgi-bin/carddisp.pl?gene=ZIC1&keywords=ZIC1> ,
