# Supplementary material for: Comparative analysis of the myoglobin gene in whales and humans reveals evolutionary changes in regulatory elements and expression levels
Source: PLoS One. 2023 Aug 29;18(8):e0284834. doi: 10.1371/journal.pone.0284834 (PMC10464968; doi:10.1371/journal.pone.0284834)
Supplement: S8 File — A Details of the clonings used for the species scan. B Published sequence accession numbers. C Primers used for clonings. D Primers and polymerase reagents used for mutations and deletions. (DOCX) [file pone.0284834.s008.docx]

**S8 File. Supporting information for Materials and Methods.**

**A** Details of the clonings used for the species scan.

*B. acutorostrata* (Ba): The Ba 5’ region was cloned twice independently. The first cloning resulted in Ba710, and used MnF704 as the 5’ primer in the round two PCR, resulting in two differences from the published sequence at the 5’ end as a result of the primer sequence. The second cloning resulted in Ba925, and used Ba-specific primers for round one and round two: round one used BaF992 as 5’ primer and BaR71 as 3’ primer; round two used BaF925 as the 5’ primer (so no changes were introduced into the 5’ end) and BaR32 as 3’ primer. Both clonings used a pIBI31 intermediate, so pIBI31 polylinker sequences are left at the 5’ end of the pGL4.10 clone.

For subsequent experiments, the Ba710x primer was used; Ba710x matches the published sequence and the Ba925 sequence, and has an XhoI site at its 5’ end, so the majority of the mutants and derivatives shown in Fig 3, Fig 4, and Fig 6 have 5’ ends that are inserted directly into the XhoI site of pGL4.10. These clones match published sequence at their 5’ ends.

Within the body of the cloned region (3’ of the primer) both the Ba710 and the Ba925 clones have 6 differences from the published sequence. Since the two clonings were independent, we conclude these differences are the correct sequence present in the genome of our Ba sample. We note that our sample was collected at Barnstable Harbor, MA, USA, whereas the published sequence is derived from a sample collected off the east coast of Korea [Yim HS, Cho YS, Guang X, Kang SG, Jeong JY, Cha SS, et al. Minke whale genome and aquatic adaptation in cetaceans. Nat Genet. 2014 Jan;46(1):88-92. doi: 10.1038/ng.2835. Epub 2013 Nov 24. PMID: 24270359; PMCID: PMC4079537.]. In addition, 4/6 of the differences are conserved in gray, blue and humpback whale sequences.

For each difference, the nucleotide differences are described with the published nucleotide first, followed by the position in the published sequence, then the nucleotide determined by our cloning: T350A (this T🡪A is conserved in Ba, Mn, Bm, and Er); C321A (C🡪A conserved in Ba, Mn, Bm, and Er); G180C (5-6 nt gaps occur in this position in Mn, Bm, and Er); G170A (A is not conserved in Mn, Bm, or Er); G167T (G🡪T conserved in Ba, Mn, Bm, and Er); G160A (G🡪A conserved in Ba, Mn, Bm, and Er).

*E. robustus* (Er): Er was cloned using BaF710x (which matches the Er sequence exactly) with a XhoI site at its’ 5’ end as the 5’ primer in the round two PCR, and is inserted directly into the XhoI and NcoI sites in pGL4.10. The sequence agrees perfectly (701/701 matches) with the published sequence.

*D. capensis* (Dc): Dc was cloned using DcF706x with a XhoI site at the 5’ end as the 5’ primer in the round two PCR, and is inserted directly into the XhoI and NcoI sites in pGL4.10. No published *D. capensis* sequence is available for comparison. However, comparison was made to published sequence from two dolphins: *Tursiops truncatus* (Tt) and *Lagenorhynchus obliquidens* (Lo). Within the body of the cloned region, three nucleotides differ from both of the other species: A(Tt)/C(Lo)255T; G176T; G173T (numbering is relative to *T. truncatus*).

*P. phocoena* (Pp): Pp was cloned using DcF706x as the 5’ primer in the round two PCR, resulting in eight primer-derived differences relative to published sequence; the product is inserted directly into the XhoI and NcoI sites in pGL4.10. Within the body of the cloned region, three differences from published sequence are found: G157T, G158del, and A138T (numbering relative to published sequence). Published *Phocoena sinus* sequence agrees with published *Phocoena phocoena* sequence at these three positions.

*B. taurus* (Bt): Bt was cloned using bosF2 as the 5’ primer in the round two PCR, resulting in two differences from the published sequence as a result of the degenerate primer nucleotides. The cloning used a pIBI31 intermediate, so pIBI31 polylinker sequences are left at the 5’ end of the pGL4.10 clone. Within the body of the cloned region, a single G449A difference from published sequence is found.

*C. canadensis* (Cc): Cc was cloned using DcF706x as the 5’ primer in the round two PCR, resulting in two primer-derived differences relative to published sequence; the product is inserted directly into the XhoI and NcoI sites in pGL4.10. Within the body of the cloned region, three differences from published sequence are found: A556C, G208A, and A167G.

*E. caballus* (Ec): Ec was cloned using Ec-specific primers: The round one PCR used EcF1 as the 5’ primer and stenR3 as the 3’ primer; the round two PCR used EcF675x as the 5’ primer and EcR51 as the 3’ primer. The product is inserted directly into the XhoI and NcoI sites in pGL4.10. In addition, instead of the Pfx polymerase, we used the high fidelity Phusion Hot Start II polymerase (Thermo, catalog #F565S) with the addition of DMSO to enhance performance on GC-rich sequences. The product is inserted directly into the XhoI and NcoI sites in pGL4.10. The sequence agrees perfectly (675/675 matches) with the published sequence.

*C. familiaris* (Cf): Cf was cloned using CF-specific primers: The round one PCR used CfF1005 as the 5’ primer and CfR65 as the 3’ primer; the round two PCR used CfF708 as the 5’ primer and CfR38 as the 3’ primer. The cloning used a pIBI31 intermediate, so pIBI31 polylinker sequences are left at the 5’ end of the pGL4.10 clone. The sequence agrees perfectly (708/708 matches) with the published sequence.

*H sapiens* (Hs): Hs was cloned using HsF671x with a XhoI site at its’ 5’ end as the 5’ primer in the round two PCR, and is inserted directly into the XhoI and NcoI sites in pGL4.10. The sequence agrees perfectly (671/671 matches) with the published sequence.

**B** Published sequence accession numbers.

*B. acutorostrata*: NW_006725354.1, REGION: 1953036..1953749 [https://www.ncbi.nlm.nih.gov/nuccore/NW_006725354.1?report=genbank&from=1953659&to=1963768]

*E. robustus*: NIPP01005289.1, REGION: 125387..126091

[https://blast.ncbi.nlm.nih.gov/Blast.cgi#alnHdr_RJWN010000952]

*D. capensis*: no reference sequence available. *T. truncatus*: NC_047044.1, REGION: 12018928..12019629[https://www.ncbi.nlm.nih.gov/gene/?term=Tursiops%20truncatus%20NC_047044.1%20myoglobin]. *L. obliquidens*: NW_020837952.1, REGION: 52920867..52921570 [https://www.ncbi.nlm.nih.gov/gene/?term=Lagenorhynchus+obliquidens+myoglobin]

*P. phocoena*: RJWQ010014833.1, REGION: 25953..26650

[https://www.ncbi.nlm.nih.gov/projects/sviewer/?RID=ZRW5MHJU013&id=RJWQ010014833.1&tracks=[key:sequence_track,name:Sequence,display_name:Sequence,id:STD1,category:Sequence,annots:Sequence,ShowLabel:true][key:gene_model_track,CDSProductFeats:false][key:alignment_track,name:other%20alignments,annots:NG%20Alignments|Refseq%20Alignments|Gnomon%20Alignments|Unnamed,shown:false]&v=25911:26685&appname=ncbiblast&link_loc=fromHSP]

*B. taurus*: NC_037332.1, REGION: 73814162..73824954

[https://www.ncbi.nlm.nih.gov/gene/280695]

*C. canadensis*: NC_057406, REGION: 57379090..57379800

[https://www.ncbi.nlm.nih.gov/gene/?term=Cervus+canadensis+NC_057406+myoglobin]

*E. caballus*: NC_009171.3, REGION: 34199488..34200166

[https://www.ncbi.nlm.nih.gov/gene/?term=Equus+caballus%3A+NC_009171.3+myoglobin]

*C. familiaris*: NC_051814.1, REGION: 29359734..29360445

[https://www.ncbi.nlm.nih.gov/gene/?term=Canis+familiaris+NC_051814.1+myoglobin]

*H. sapiens*: NG_007075.1, REGION: 10427..11101

[https://www.ncbi.nlm.nih.gov/gene/?term=H.%20sapiens%20NG_007075.1%20myoglobin]

**C** Primers used for clonings (5’ to 3’).

BaF992: GTGCAACCATGGCAGATTTCC

BaR71: CCTGCGACATCAGCTTCCAC

BaF925: AGGGCTGTTGGGGGACTAA

BaR32: ACCAAGTGCCATTCTGCGTC

BaF710x: ccctcgagTCCAGCAAAACTTTTCAGAGAG

bosF1: RGTTCAARWCCCARCTCTKCC

bosF2: TSAMGMAMAAYTTTTCAGAGG

CfF1005: CTTGGCATGTAGCAGTTTCC

CfR65: AGGTCAGTCTCCACCTTC

CfF708: TCAAGCAAAGCTTCTCAGAAG

CfR38: TTCAGCACCAACTGCCATTC

DcF706: TGACGCACAATTTTTCAGAGG

DcF706x: ccctcgagTGACGCACAATTTTTCAGAGG

EcF1: GGTTCAAGTCCCAGCTCTTCCG

EcF675x: ccctcgaGTCAAGCAAAACTTTTCAGAGGG

EcR51: CTTCCCCCAGACGTTCAGCA

HsF671X: ccctcgaGTCAAGAAAAACTTTTCAGAGG

MnF704: TCACGCAAAACTTTTCAGAGAG

stenR1: CATGGCACAGTCTGAAGA

stenR2: cttcccccagacgttcagta

stenR3: agatcagcctccaccttc

**D** Primers and polymerase reagents used for mutations and deletions.

Ba MEFmut: (Q5 Site-Directed Mutagenesis Kit (NEB))

BaFmef: gcttCTTCCATGTGAGGGCCAG

BaRmef: gcgcAGGCAGGTGCCATTGTGG

Ba ΔAT: (QuikChange II Site-Directed Mutagenesis Kit (Agilent))

ΔATF: ACCACCCCACAATGGgaattcAGGGCCAGAGAAATG

ΔATR: CATTTCTCTGGCCCTgaattcCCATTGTGGGGTGGT

ATswap: (Q5 Site-Directed Mutagenesis Kit (NEB))

ATswapF: GCTTcCCATGTGAGGGCCAGAGAAATGAAAAG

ATswapR: TATTTTagGGCAGGTGCCATTGTGGG

Ba E-box1mut: (Q5 Site-Directed Mutagenesis Kit (NEB))

BaFebox1: CCCACAATGGgaattcCCTCAAAATAGCTTCC

BaRebox1: GTGGTAGGACAACTCAGG

Ba E-box2mut: (Q5 Site-Directed Mutagenesis Kit (NEB))

BaFebox2: tcaGGGCCAGAGAAATGAAAAG

BaRebox2: attcGAAGCTATTTTGAGGCAG

Ba E-box3mut:

BaFebox3: gggaattcTCGGAGCCAGGACAC

BaRebox3: gggaattcGGGTTTGAGGCTGCCT

Ba CCACmut: (Q5 Site-Directed Mutagenesis Kit (NEB))

BaFccac: GCTCCTCCCCggtaCCCCAACCTG

BaRccac: TGTGTGTGTGGAAAGAGTG

Ba ΔCCAC: (QuikChange II Site-Directed Mutagenesis Kit (Agilent))

ΔCCACF: ACCTCCTCCCCCCTCgaattcGGCACCTGCCTCAAAA

ΔCCACR: TTTTGAGGCAGGTGCCgaattcGAGGGGGGAGGAGGT

Ba ΔSP1-CCAC:

BaR335e: gggaattcgatcaccgtctgggctc

CCACswap: (Q5 Site-Directed Mutagenesis Kit (NEB))

CCACswapF: ccccctgtggcCTGAGTTGTCCTACCACC

CCACswapR: tggggtggttgtGTGTGTGGAAAGAGTGGG

Ba410:

BaF410: ccctcGAGTTGGGTTTCAGGCC

Hs ΔAT: (QuikChange II Site-Directed Mutagenesis Kit (Agilent))

HsΔATF: CTCGCCACAATGGgaattcAGGGCTAGAGAAAG

HsΔATR: ctttctctagccctgaattcccattgtggcgag

Hs ΔCCAC: (QuikChange II Site-Directed Mutagenesis Kit (Agilent))

HssΔCCACF: CAACCTCCTCCCCTTCgaattcGGCACCTGCCCTAA

HsΔCCACR: TTAGGGCAGGTGCCgaattcGAAGGGGAGGAGGTTG

Hs ΔG-rich: (Q5 Site-Directed Mutagenesis Kit (NEB))

HsF3xsp1: cagACAGCGAGCCATTGAGCG

HsR3xsp1: cagCTCTCTCATCCAGGGAGGG

HsF4xsp1: cagTGGATGAGAGAGCTGCAG

HsR4xsp1: cagTCTAATCTTTTCCTTTCTCTAGC

Ba ΔG-rich:

BaR181: cccctcgaGCTTTCATGCAGGG

BaF155: ccctcGAGAGAGAGAGTGAGCGAGC

Ba Δ460/411:

BaF410e: gggaattcGAGTTGGGTTTCAGGCC

BaR461e: gggaattCAGCAGTCACCCTGG

Ba 3kb region:

3kbF2: GGACGTGTAGTCTGGACAGGA

3kbR3.2: ACAGAAACGAAGGCTTGGAG

3kbF3: GACAAGGGAGTGAGCGTGAC

3kbR3.1: AAACGAAGGCTTGGAGAGGT
