# Supplementary material for: Comparative analysis of the myoglobin gene in whales and humans reveals evolutionary changes in regulatory elements and expression levels
Source: PLoS One. 2023 Aug 29;18(8):e0284834. doi: 10.1371/journal.pone.0284834 (PMC10464968; doi:10.1371/journal.pone.0284834)

**S9 File. Differentiation of C2C12 cells under transfection conditions.**

Differentiation of C2C12 cells over 4 days in differentiation medium. Top row: Phase contrast pictures of transfected cells. Bottom row: Untransfected cells stained for myosin heavy chain; primary antibody was mouse monoclonal anti-myosin (skeletal, slow) from Sigma-Aldrich (catalog # M8421, RRID:AB_477248) at 1:1000 dilution; detection with horse radish peroxidase used Vector ImmPress kit (catalog # MP-7402).


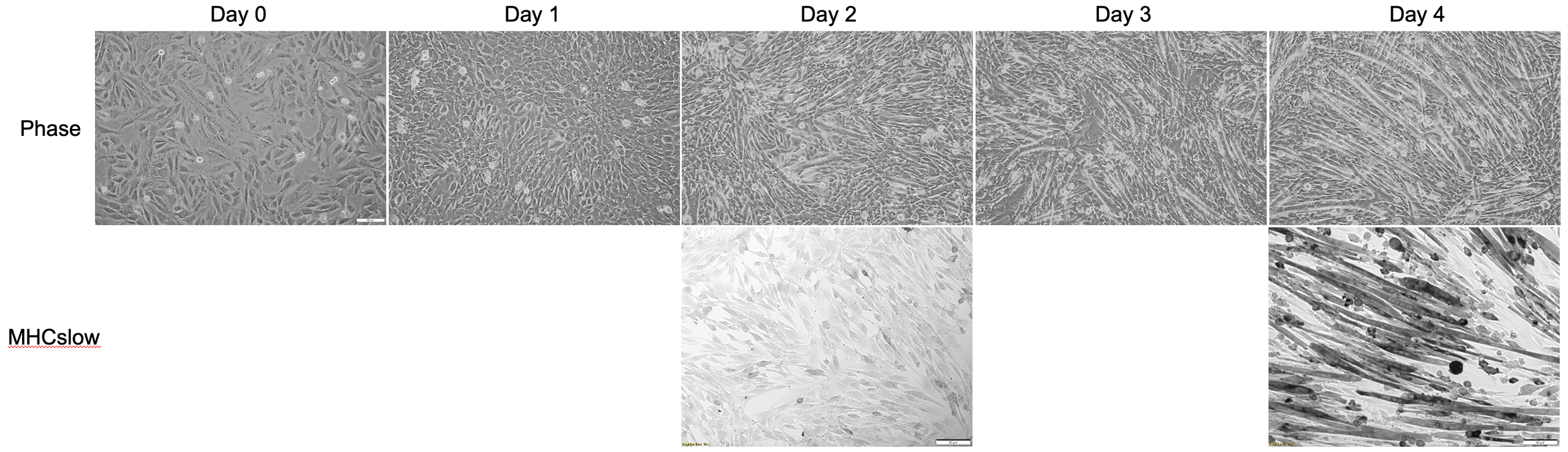

Supplement: S9 File — Differentiation of C2C12 cells over 4 days in differentiation medium. Top row: Phase contrast pictures of transfected cells. Bottom row: Untransfected cells stained for myosin heavy chain; primary antibody was mouse monoclonal anti-myosin (skeletal, slow) from Sigma-Aldrich (cat # M8421, RRID:AB_477248) at 1:1000 dilution; detection with horse radish peroxidase used Vector ImmPress kit (cat # MP-7402). (DOCX) [file pone.0284834.s009.docx]
