# Supplementary material for: Comparative analysis of the myoglobin gene in whales and humans reveals evolutionary changes in regulatory elements and expression levels
Source: PLoS One. 2023 Aug 29;18(8):e0284834. doi: 10.1371/journal.pone.0284834 (PMC10464968; doi:10.1371/journal.pone.0284834)
Supplement: Attachment — Submitted filename: Comments to the authors.docx [file pone.0284834.s011.docx]

Comments to the authors:

This paper describes their studies on transcription control of myoglobin (Mb) gene in cetacean and comparison of the mechanisms with human. They started the investigation by hypothesizing the higher Mb concentrations in cetacean myocytes, which are required for their deep-diving capacity, may be due to high transcription rates of the gene. However, their experiments with cultured recombinant cells indicated that basal level of the transcription rates in cetacean's expression system is lower than that in human's system. Thus, they concluded that the higher Mb concentrations in cetaceans' myocytes are not due the constitutive transcription rates but mainly due to protein stability of the Mb molecules. On the other hand, Mb transcription of horse with higher Mb protein level is much stronger than human, according to their present results. The reviewer's main question is why cetacean did take strategy to improve the protein stability (and solubility) rather than to improve the transcription rates. The authors should also consider recent studies concerning molecular evolution of diving mammal Mbs and discuss this problem furthermore. The references that were not cited in the present manuscript are follows:

Y. Isogai, H. Imamura, T. Sumi and T. Shirai, Improvement of protein solubility in macromolecular crowding during myoglobin evolution. *Biochemistry* 61, 2022, 1543–1547, 2022.

Y. Isogai, H. Imamura, S. Nakae, T. Sumi, K. Takahashi, T. Nakagawa, and T. Shirai, Common and unique strategies of myoglobin evolution for deep-sea adaptation of diving mammals**.** *iScence* 24(8): 102920, 2021

Berenbrink, M. The role of myoglobin in the evolution of mammalian diving capacity - The August Krogh principle applied in molecular and evolutionary physiology. *Comp Biochem Physiol A Mol Integr Physiol* 252, 110843, 2021.

Y. Isogai, H. Imamura, S. Nakae, T. Sumi, K. Takahashi, T. Nakagawa, A. Tsuneshige and T. Shirai, Tracing whale myoglobin evolution by resurrecting ancient proteins. *Sci. Rep.*8*,* Article number: 16883, 2018

Minor points:

The authors have investigated the Mb expression of baleen and small toothed whales. However, diving capacity of these whales is generally lower than large toothed whales such as sperm whale, and the Mb concentrations are likewise (Noren & Williams, Comparative Biochemistry and Physiology Part A 126, 181-191, 2000; Helbo et al., J Exp Biol 215, 3403–3410, 2012). This point should be addressed.
