# Supplementary material for: Comparative analysis of the myoglobin gene in whales and humans reveals evolutionary changes in regulatory elements and expression levels
Source: PLoS One. 2023 Aug 29;18(8):e0284834. doi: 10.1371/journal.pone.0284834 (PMC10464968; doi:10.1371/journal.pone.0284834)
Supplement: Attachment — Submitted filename: Comments__230603.docx [file pone.0284834.s012.docx]

Comments for the manuscript entitled “Comparative analysis of myoglobin in Cetaceans and humans reveals novel regulatory elements and evolutionary flexibility” prepared for PLOS ONE.

The authors analyzed the promoter regions of myoglobin genes of Cetaceans and land animals species mainly by deleting or substituting identified elements such as CCAC box or AT element.  They found that the functionalities of the elements were largely different between Cetaceans and some of others, and proposed CCAC box did not contribute for Mb expression in Cetaceans, but G-rich region 3’ of the AT element  had positive effect instead. They also identified novel upstream element as an enhancer. The purpose of the presented research sounds and results have been mostly appropriately presented. However, this reviewer could not be positive to recommend this paper for publication in its current form. The opinion of this reviewer would be reconsidered if the problems in the current manuscript, as mentioned in the attached letter, are satisfactory addressed by the authors.

Major points:

1) To this reviewer’s opinion, the most critical disadvantage of the current study should be as follows. The authors mentioned that one of the weak points was missed *cis*-acting elements. However, the most critical point should be *trans*-acting elements including transcription factors and basic transcription machineries. The authors expressed the reporter gene in C2C12 in cells. Therefore, it just showed, for example, mink whale CCAC box was inactive in the mouse background (TF, RNA pol, etc…), and functionality of this element would be different between mouse and whale. It could add new information, but demonstrated nothing about the role of the element in the whale. The authors should reasonably address this problem and explain rationales in the text. To this reviewer, this point is mandatory for reconsidering the decision for recommendation.
2) The authors did ANOVA followed by the Tukey test independently for each presented set of samples by selecting (without explicit criteria) one of the samples as a reference and scaled to 1.0. This reviewer did not think this procedure had a problem, but felt difficulty in interpreting the presented results when comparing between different sets (Figures). It would be possible that (a) uniformly select wild-type (or another appropriate one) as the reference and scale it to 1.0 for all presentations, and (b) apply multiple comparison procedure for all pair of the samples in the presentation, and (c) show significantly deviated pairs with P-values. This reviewer strongly recommends this statistical presentation.

Minor points:

3) The title of this paper “Comparative analysis of myoglobin in Cetaceans and humans reveals novel regulatory elements and evolutionary flexibility” was uncomfortable to this reviewer. What are “humans”? Are they human and other land animals? It sounds like homo sapiens and homo neanderthalensis …, for example.
4) This reviewer noticed several typos such as “element ar edispensable” (page 39). They should be fixed.
5) Page 39 “functional swap”. This reviewer could get the nuance of this term, but its meaning should be explicitly defined in the text.
6) Figure 7B: This reviewer knows many researchers do this, but using screenshots of genome browser for a Figure (as it is) is not recommended, because they are too busy. The authors should retouch the Figure.
