# Supplementary material for: Comparative analysis of the myoglobin gene in whales and humans reveals evolutionary changes in regulatory elements and expression levels
Source: PLoS One. 2023 Aug 29;18(8):e0284834. doi: 10.1371/journal.pone.0284834 (PMC10464968; doi:10.1371/journal.pone.0284834)
Supplement: Attachment — Submitted filename: Response to reviewers.docx [file pone.0284834.s013.docx]

Response to reviewers:

Academic editor:

- Re: Trans-acting elements: This point was raised by the reviewer of “Comment_230603” and has been addressed as described in the response to that reviewer, below.
- Re: More references: Suggested references were provided by two reviewers. The reviewer of the “Comments to the authors” (reviewer #2) suggested papers by Isogai et al and Berenbink, and these have been incorporated as described in the response to that reviewer, below. Also, reviewer #3 suggested papers by Cartwright and Noren; these have been included as described in the response to that reviewer, below.
- Re: The potential linkage between the promoter activity of the whale myoglobin and the slow myoglobin accumulation in cetaceans: This point is raised by reviewer #3 and has been addressed as described in the response to that reviewer, below.
- Re: The pros and cons of improving transcription rate or protein stability: This issue, too, is raised by reviewer #3. The comments of reviewer #3 have led to considerable revision of the Discussion section titled “Transcription driven by minke whale 5’ flanking regions is only 8% that of humans”. We originally had discussed “post-transcriptional mechanisms”, which would imply regulatory mechanisms operating in real time in response to conditions (e.g., increases in mRNA stability or in translational rate). Instead, reviewer #2 and #3’s comments have steered us toward protein evolution. In our opinion this has greatly improved this portion of the discussion, and better addresses some of the thinking in the field that we had not given sufficient attention.

Re: Journal Requirements:

- The initial review indicated that the style requirements have been followed.
- 2, 3. The wording in the Acknowledgements section was not intended to imply funding. The wording has been changed. The Funding statement is not changed. The new sections are in the cover letter.
- 4. Regarding data availability, all data has been included in the manuscript and supplemental files, as stated in the Data Availability section. S10 File has been added to include the data and an example of how raw data was processed in an accessible Excel format.
- 5. Supporting information files have been listed at the end of the manuscript. Included are the name in the form of “S1 File.docx”, a title, and a legend.

Additional notes:

Two changes to the submitted material have been made that are not in response to the editors or reviewers’ comments:

- S4 File: A Tukey test comparing Ba ∆AT, Ba ∆CCAC, and Ba ∆CCAC-AT has been added to S4C. Previously, a T-test was used to determine if Ba ∆AT and Ba ∆CCAC-AT were significantly different, from which a slight degree of significance (p = 0.04) was concluded. However, in exploring this question using a Tukey test on various data sets, in no case was significance indicated. We therefore felt it was most appropriate to analyze a 3-way comparison with a Tukey test, and provide that as evidence that Ba ∆AT and Ba ∆CCAC-AT are not, in fact, significantly different.
- Fig 4C: One value had been entered wrong in producing the graph; this value has been corrected and is reflected in Fig 4C, S4D File, Fig 4 legend, and relevant text. This correction does not change the conclusion that Ba410 and Ba410∆CCAC are not significantly different.

Response to “Comments_230603”:

Major points:

1) Response:

This issue is addressed in two places:

In the Discussion/Transcription driven by minke whale 5’ flanking regions is only 8% that of humans/paragraph 5: We point out that mouse C2C12 have been widely used for many years, but discuss and reference work that supports the possibility that they may not be a perfect model system for whale studies, due to possible variations in transcription factor activity from other commonly used model systems and from the in vivo situation.

In the Discussion/Limitations of the study/paragraph 3: We again raise this issue, and note that the same criticism can be made for the human CCAC-box in that its definition was also done in non-human model systems. Repeating experiments designed to define the CCAC-box in human and (as yet unavailable) cetacean muscle-cell culture systems would be of interest.

We also discuss mechanisms through which the activities of the minke and human CCAC-boxes may differ in the Discussion/The minke whale gene has substituted a G-rich sequence for the CCAC-box/paragraphs 7 and 9 (“enhancer grammar”, and possible binding of MYOD to the human but not minke CCAC-box).

2) Response:

We have chosen to use an experimental sample with an activity very close to “1.0” for the statistical comparisons in each set (figure); in this way we avoid always using the same wild-type Ba710 data due to the statistical problems this would have caused. In each set (figure) there was always one or more samples that could represent the null hypothesis: that is, the mutation does not have an effect on expression. We appreciate that this can cause confusion when comparing different sets of experiments, but each figure is scaled to the activity of wild-type Ba710 = 1.0 by the data analysis, which is now described explicitly in S10 File. We felt this was the least problematic approach to the data.

So, addressing point (a), each sample was normalized in two steps: firefly luciferase counts in each well was normalized to an internal renilla luciferase standard included in the transfection to control for well-to-well variation, and each plate was normalized to wild-type Ba710 = 1.0 to control for plate-to-plate variation. The result is that on the Y-axis in each graph the 1.0 value is that of the wild-type Ba710 construct in each particular transfection plate.

Regarding point (b), the Tukey test does compare each sample against each other sample, and a summary of the Tukey test is shown in the Supplemental data files. The authors felt that including all of the comparisons (e.g., A vs B, A vs C, A vs D, B vs C, B vs D, and C vs D) was confusing, and it would be clearest to only present the chosen control against each of the others. We have added a Supplemental file (S10 File) to address your concern with this point. It contains all the data to allow readers to test the various possible comparisons; the samples designated as “significantly different” are generally highly significant, and so are quite robust to this kind of playing around with the data. S10 File also contains an example of how raw luciferase counts were converted to the “F/R/Ba” number so the reader can better understand where the value of 1.0 comes from.

For point (c), the actual P-values are stated in the legend to each figure. The authors felt that showing asterisks on the graph itself rather than P-values would be clearer for the reader.

Minor points:

3. We appreciate that the title was a bit awkward. Perhaps part of the problem was caused by mixing the technical term “cetacean” with the colloquial term “human”? We have changed it to “Comparative analysis of the myoglobin gene in whales and humans reveals evolutionary changes in regulatory elements and expression levels”. We hope this is better.

4. The manuscript has been carefully read and subjected to Word spell- and grammar-checks. We believe we have caught all the typos.

5. We have changed the “functional swap” comment in the Discussion/ The minke whale gene has substituted a G-rich sequence for the CCAC-box/paragraph 8 to “Sequence flexibility in these runs of G-C nucleotides may have facilitated the proposed evolutionary switch between the CCAC-box and the G-rich sequence.” We hope this clarifies the meaning.

6. We appreciate that our original Fig 7B was complicated, so we have simplified it to make it more accessible. A link in the legend will take the reader to the original at the Genome Browser site for those who are interested.

Response to “Comments to the authors”:

Major point:

The authors thank this reviewer for their insightful comments and suggestions of references that were not included. A fresh consideration of the work of Isogai et al has changed our perception of the significance of our own work, and we have changed our discussion accordingly (Discussion/Transcription driven by minke whale 5’ flanking regions is only 8% that of humans/paragraphs 7 and 8). We had previously focused on regulatory mechanisms operating in real time (increasing translation or protein stability), rather than the myoglobin protein evolution that has occurred.

We briefly review in this section the evolution of stability and solubility of the MB protein and in this way better include the work of Isogai et al and Berenbink, and address the question of “why cetacean did take strategy to improve the protein stability (and solubility) rather than to improve the transcription rates”. We conclude that “Therefore, high transcription levels are not necessary to account for high MB protein levels in adult cetacean animals” and we note that “Evolving an increased rate of transcription to satisfy the physiological need for high MB levels in the absence of protein evolution would not have solved the aggregation problem. Evolving instead a stabilized protein resistant to aggregation is a strategy widely shared among diving mammals.”

Minor point:

The tissue samples we had at our disposal were primarily representative of the mysticetes due to the history of this work (Cartwright et al. 2016. Tracking the development of muscular myoglobin stores in mysticete calves. PLoS One, 11(1), p.e0145893). We did have dolphin and porpoise available but did not have sperm whale samples.

Due to the consistently low levels of transcription from the seven species tested, as well as the related terrestrial species (cow, elk, pig), expanding our survey did not seem to be a priority.

The point is well taken, however, and perhaps future work could include sperm whales and beaked whales (probably the diving world record holders). It would also be of interest to include Weddell seals in a direct comparison to whales, since seals are such a distant group of diving mammal.

Response to Reviewer #3:

The authors thank this reviewer for their suggestion of references to include, and we have done so. In the Discussion/Transcription driven by minke whale 5’ flanking regions is only 8% that of humans/paragraph 7 we point out that myoglobin levels increase steadily throughout the life of the animal, and reference these papers. In response to the linkage between low transcription levels and high adult protein levels, we conclude that, due to the evolved stability of the protein, high transcriptional rates are not required for the eventual accumulation of high protein levels.

The last two “minor points” are also relevant to this issue and the authors thank this reviewer for bringing them up as well. One of the other reviewers has pointed us to the work of Isogai et al and Berenbink regarding the evolution of the myoglobin protein toward greater solubility, more stable folding, and resistance to aggregation in diving mammals. We therefore propose that the evolutionary “reasoning” behind low transcription levels is that high transcription levels without this concomitant protein evolution would lead to myoglobin aggregation and destruction, and would therefore be counterproductive, as this reviewer has pointed out. Again, due to the increased stability of the protein, slow and steady does the job.

In a similar vein, one may speculate that the evolution of prolonged maternal care and maternal coaching of energetic behaviors such as breaching may play into this issue. The induction of increased transcription by signaling mechanisms accompanying such behavior may be able to be titrated by the mom-coach for an optimal tradeoff between expression levels and protein accumulation. This is outside the scope of this particular paper, however.

We have also removed the implication that low transcription and protein stability “co-evolved”. The reviewer is absolutely correct in pointing out that the low transcription levels are most likely to be ancestral, and we raise this point in paragraph 8 of this section. It improves the discussion, and thank this reviewer for bringing it up.

Other minor points:

Thank you for the correction regarding capitalization of Cetacea and cetaceans. This has been corrected.

The sentence “Traditionally, evolution has focused on changes in amino acid sequence” has been changed to read: “Traditionally, the study of evolutionary change has focused on changes in amino acid sequence…” We hope this is clearer.

“the Cetacea” has been corrected to “baleen whales.

The manuscript has been carefully read for typos, and run through Word spell check. We believe we have corrected all typos.
